# A Novel Variable Selection Method Based on Ordered Predictors Selection and Successive Projections Algorithm for Predicting Gastrodin Content in Fresh *Gastrodia elata* Using Fourier Transform Near-Infrared Spectroscopy and Chemometrics

**DOI:** 10.3390/foods12244435

**Published:** 2023-12-11

**Authors:** Zhenjie Wang, Changzhou Zuo, Min Chen, Jin Song, Kang Tu, Weijie Lan, Chunyang Li, Leiqing Pan

**Affiliations:** 1 College of Food Science and Technology, Nanjing Agricultural University, No. 1 Weigang Road, Nanjing 210095, China; 2021208018@stu.njau.edu.cn (Z.W.); 2021208019@stu.njau.edu.cn (C.Z.); 2019208017@njau.edu.cn (M.C.); kangtu@njau.edu.cn (K.T.); weijie.lan@njau.edu.cn (W.L.); 2 College of Artificial Intelligence, Nanjing Agricultural University, No. 40 Dianjiangtai Road, Nanjing 210095, China; 2020212019@stu.njau.edu.cn; 3 Institute of Agro-Products Processing, Jiangsu Academy of Agricultural Sciences, No. 50 Zhongling Road, Nanjing 210014, China

**Keywords:** *Gastrodia elata*, gastrodin, near-infrared spectroscopy (NIRS), partial least squares (PLS), multiple linear regression (MLR)

## Abstract

Gastrodin is one of the most important biologically active components of *Gastrodia elata*, which has many health benefits as a dietary and health food supplement. However, gastrodin measurement traditionally relies on laboratory and sophisticated instruments. This research was aimed at developing a rapid and non-destructive method based on Fourier transform near infrared (FT-NIR) to predict gastrodin content in fresh *Gastrodia elata*. Auto-ordered predictors selection (autoOPS) and successive projections algorithm (SPA) were applied to select the most informative variables related to gastrodin content. Based on that, partial least squares regression (PLSR) and multiple linear regression (MLR) models were compared. The autoOPS-SPA-MLR model showed the best prediction performances, with the determination coefficient of prediction ($R_{p}^{2}$), ratio performance deviation (RPD) and range error ratio (RER) values of 0.9712, 5.83 and 27.65, respectively. Consequently, these results indicated that FT-NIRS technique combined with chemometrics could be an efficient tool to rapidly quantify gastrodin in *Gastrodia elata* and thus facilitate quality control of *Gastrodia elata*.

## 1. Introduction

*Gastrodia elata* Blume (*G. elata*, Orchidaceae), a traditional Chinese medicine (TCM) named ‘Tianma’, is widely used as a dietary product and nutritional supplement because of its valuable bioactive compounds [1]. Currently, over 81 compounds, encompassing phenols, polysaccharides, sterols, and organic acids, have been isolated and identified from *G. elata* [2]. Particularly, gastrodin (GAS) represents a primary phenolic compound found in *G. elata*. In recent years, intensive research on GAS has revealed a diverse array of biological activities, including anti-tumor, antiviral, memory enhancement, antioxidative, and anti-aging effects [3,4,5].

Currently, durable ecological and climatic factors from different producing regions can result in a large variability of GAS in *G. elata*, leaving the increasing market demands unsatisfied. Currently, the quantification of gastrodin (GAS) in *G. elata* typically involves chromatographic techniques, such as high-performance liquid chromatography (HPLC) and gas chromatography–mass spectrometry (GC-MS) [3]. Those methods provide accurate determinations of GAS but suffer from the drawbacks of time-consuming, destructive, and complex sample pre-treatments. Therefore, developing rapid, non-destructive, and economical methods to detect GAS content in *G. elata* could help producers and industrial manufacturers to better manage their quality.

Near-infrared spectroscopy (NIRS) relies on the absorption of electromagnetic radiation within the wavelength range spanning from 780 to 2500 nm (14,286–4000 cm^−1^) [6]. It is characterized by low molar absorption and scattering, allowing for rapid, non-destructive analysis of samples without requiring sample preparation [7]. In the present day, NIRS has evolved into a potent tool for swiftly quantifying chemical compounds in plants [8]. For example, atractylenolide-1 is the most important bioactive ingredient in *Rhizoma atractylodis macrocephalae*, which can be well determined by NIRS coupled with partial least squares (PLS) algorithm [9]. Furthermore, the contents of polysaccharides, flavonoids, and alkaloids in several plant-based products have been successfully analyzed by NIRS [10,11,12].

Establishing a reliable and robust calibration model constitutes a crucial step in the application of NIRS [13]. The intrinsic complexity of spectra, characterized by large sets of predictor variables, irrelevant information, and noise, poses a challenge to calibration in NIRS analysis. To mitigate these effects, optimization of various parameters, including spectral preprocessing, variable selection, and potential factors, becomes essential. Numerous studies have demonstrated that more accurate calibration models can be achieved by selecting informative spectral variables instead of utilizing the full spectra. Introducing only noisy features is found to deteriorate rather than improve model performance [14]. Variable selection approaches play a pivotal role in spectral data analysis by identifying a subset of relevant variables from an initial set of primitive variables that carry the most important information with minimal noise [15]. Various approaches, as outlined in [16], exist for selecting an optimal subset of spectral wavelengths or regions during the calibration process. The success of spectral variable selection is typically evaluated empirically through statistical analysis of the resulting multivariate models.

This study aimed to pioneer the development of a high-throughput, rapid, nondestructive, and reliable method for quantifying GAS concentration in fresh *G. elata* using NIRS and chemometrics. In order to achieve robust and optimal calibration specificity, we employed the ordered predictors selection (autoOPS) [17], successive projections algorithm (SPA) [18] and their combination (autoOPS-SPA) to select informative variables for model development. Subsequently, PLS and multiple linear regression (MLR) models were constructed, and their prediction abilities for GAS concentration in fresh *G. elata* were compared.

## 2. Materials and Methods

### 2.1. G. elata Samples

*G. elata* samples were acquired from a commercial farm after seven-month harvest cycles, in Tongren, Guizhou province, China (108.12° N 28.27° E), on 16 November 2021. To prevent moisture loss, fresh *G. elata* samples were airlifted from Tongren to Nanjing immediately after harvest. Each sample was rinsed three times with tap water to remove soil and dirt, wiped dry with a clean towel. A total of 265 *G. elata* samples were selected in order to achieve as much uniformity as possible in appearance (i.e., size, shape and color) and reduce the variations among the test *G. elata* at the onset of the experiment. All selected samples were absent of defects or disease, and the weight was between 70 and 80 g. Afterward, the samples were analyzed by FT-NIR.

### 2.2. FT-NIR Spectroscopic Acquisition

The spectral data of *G. elata* samples were acquired using an Antaris II FT-NIR spectrophotometer (Thermo-Fisher, Waltham, MA, USA) equipped with an integrating sphere diffuse reflection system, a high sensitivity InGaAs detector, and a tungsten lamp (20 W) for diffuse reflectance measurement. Prior to spectral scanning, all samples were acclimatized in the instrument room at 20 ± 1 °C and 60 ± 1% humidity for 30 min. *G. elata* samples were positioned steadily on the fruit holder with equidistant horizontal alignment. Source fibers irradiated the samples with light, and the reflected light back from the samples was captured by detector fibers and conveyed to the FT-NIR spectrometer. Air measurements were taken at 1 h intervals as a reference background according to the instrument’s instructions.

The recorded FT-NIR spectra spanned the range of 10,000–4000 cm^−1^ (1000–2500 nm) with a spectral resolution of 2 cm^−1^ and 32 scans, resulting in 3112 spectral variables per spectrum. For each *G. elata* (Figure S1), spectra were measured at three equidistant positions around the terminal bud at room temperature (20 ± 1 °C). The averaged spectrum per *G. elata* was utilized for analysis as the original spectrum of the sample. Reflectance spectra were converted to relative reflectance by dividing each sample spectrum by the standard reference spectrum. Each spectrum was recorded as absorbance value (log (1/R)), where R = reflectance, obtained by averaging 32 scans. In the modeling, all samples were divided into a calibration set and a prediction set in the ratio of 3:1 using the Kennard–Stone (KS) algorithm based on Euclidean distance [19].

### 2.3. Gastrodin Content Measurement

The extraction and quantification of GAS were carried out according to the method by Shen et al. [20]. Briefly, fresh *G. elata* was cut into small pieces. Then, the samples were ground into powder in liquid nitrogen for GAS extraction. Next, 2.000 g of prepared sample powder and 25.0 mL of 70% methanol aqueous solution (*v*/*v*; methanol, chromatographic grade; ultrapure water) were mixed in a volumetric bottle. The mixture was then ultrasonically extracted with a 40 kHz frequency for 20 min at room temperature. After that, 10 mL was precisely pipetted into an evaporation dish and water-bathed at 70 °C until nearly dry. The residue was dissolved in the mobile phase and subsequently transferred to a 10 mL volumetric flask. The resulting mixture underwent filtration through a 0.45 μm membrane filter for subsequent analysis.

**Figure 1:**
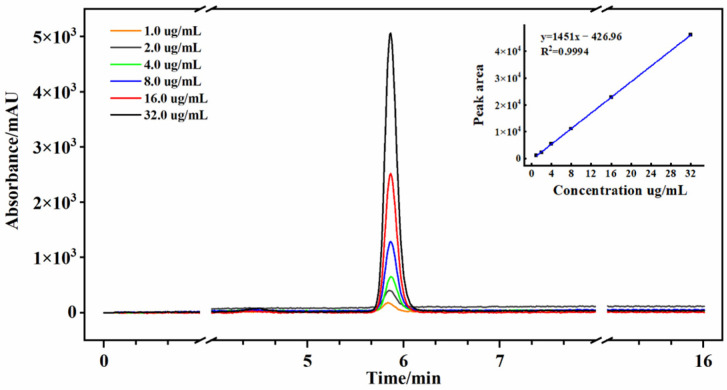
HPLC chromatogram of the standard solution.

The determination of GAS was conducted utilizing a high-performance liquid chromatography (HPLC) system equipped with a photodiode array detector (PDA) (Shimadzu LC-20A, Kyoto, Japan). Separation of compounds was achieved using a Symmetry C18 column (size: 4.6 × 250 mm, Waters, Ireland) with isocratic elution at a flow rate of 1.0 mL min^−1^. The column oven temperature was set at 30 °C, and the detector wavelength was set at 220 nm. The mobile phase consisted of a 5% acetonitrile aqueous solution (*v*/*v*, acetonitrile, chromatographic grade; ultrapure water), and 20 μL of extracts were injected into the HPLC system. Each run was completed within 16 min. The GAS standard (Sigma, St. Louis, MA, USA) was used for the establishment of calibration curves [20]. Precisely, 8.0 mg of GAS standard was added in a 50 mL volumetric flask, followed by addition of acetonitrile/water (5:95), and it was shaken properly to obtain a 160 ug mL^−1^ solution of the standard. The GAS standard diluted to the concentration ranges of 1.0, 2.0, 4.0, 8.0, 16.0 and 32.0 μg/mL was used for the establishment of calibration curves. The standard solutions were measured under the same chromatographic conditions, and the produced calibration linear equation was y = 1451.8x − 426.97, R^2^ = 0.9994. The chromatogram of standard compounds is shown in Figure 1. The peak area and content of GAS were calculated by an HPLC auto-integrator and calibration curves equation, respectively. Figure S2 presents the chromatogram of the *G. elata* sample.

### 2.4. Outlier Detection

**Figure 2:**
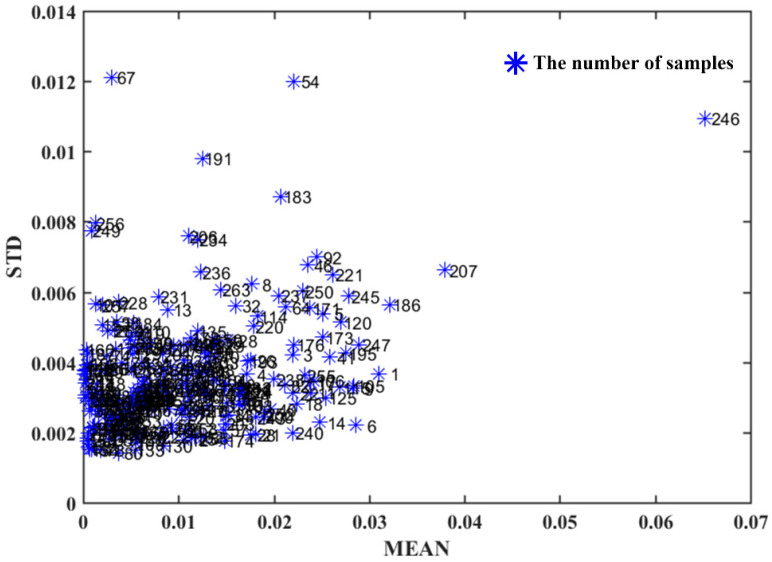
Monte Carlo outlier sample detection results.

Identifying potential outliers in the data is a crucial preliminary step, as preprocessing and multivariate analysis techniques can be significantly impacted by their presence. The Monte Carlo method was employed to detect possible outliers in the dataset [21]. During each iteration, a proportion of the samples was designated as the training set to build a model, which was then used to predict the remaining test samples. By repeatedly recording the prediction error of each test sample, and assuming each sample has an equal probability of being selected, the sample was selected into the test set for approximately 500 times, resulting in prediction errors. To facilitate outlier detection, a diagnostic map was generated by plotting the standard deviation (SD) against the mean (MEAN) of the prediction errors for each sample. Samples with elevated MEAN and/or SD values were identified as outliers. In the dataset, three samples (No. 54, 67, and 246) exhibiting high mean or standard deviation of prediction error were excluded as detected outliers (Figure 2).

### 2.5. Chemometrics and Modeling Evaluation

#### 2.5.1. Spectra Pre-Processing

Before establishing the calibration models, it is essential to meticulously preprocess the raw data. This involves implementing mathematical procedures to correct and enhance spectra. Typically, smoothing algorithms are employed to diminish systematic and random noise, thereby enhancing the spectral signal-to-noise ratio. Inevitably, fluctuations in the spectral baseline and reduced spectral resolution may arise due to the physical properties of the sample, light scattering, and differences in the effective path length. Derivatives (1st Derivative, 2nd Derivative) can effectively mitigate the baseline effect and resolve overlapping peaks. Concurrently, spectral intensity variations are rectified through standard normal variate (SNV) and multiplicative scatter correction (MSC) [22]. Employing appropriate pre-processing methods can efficiently filter spectral noise and retain valid information, thereby simplifying quantitative models and enhancing the robustness and predictability of the model. On this account, a set of spectral preprocessing methods were applied independently and combined, including mean center (MC), Savitzky–Golay (SG) smoothing, autoscale, 1st Derivative, SNV, and MSC.

#### 2.5.2. Variable Selection

The FT-NIR spectra of *G. elata* samples comprise thousands of variables, adding complexity to the analytical challenge. From a practical point of view, eliminating redundant and uninformative variables is highly desirable. On the one hand, a model consisting of numerous variables may suffer from overfitting, potentially leading to complexity in the model. On the other hand, variables with noise may result in a confusing or misleading model. Variable selection involves extracting pertinent information from an initial set of original variables for subsequent modeling. The exclusion of irrelevant and nonlinear variables serves to simplify the model, enhancing both its performance and data interpretability [23].

**Figure 3:**
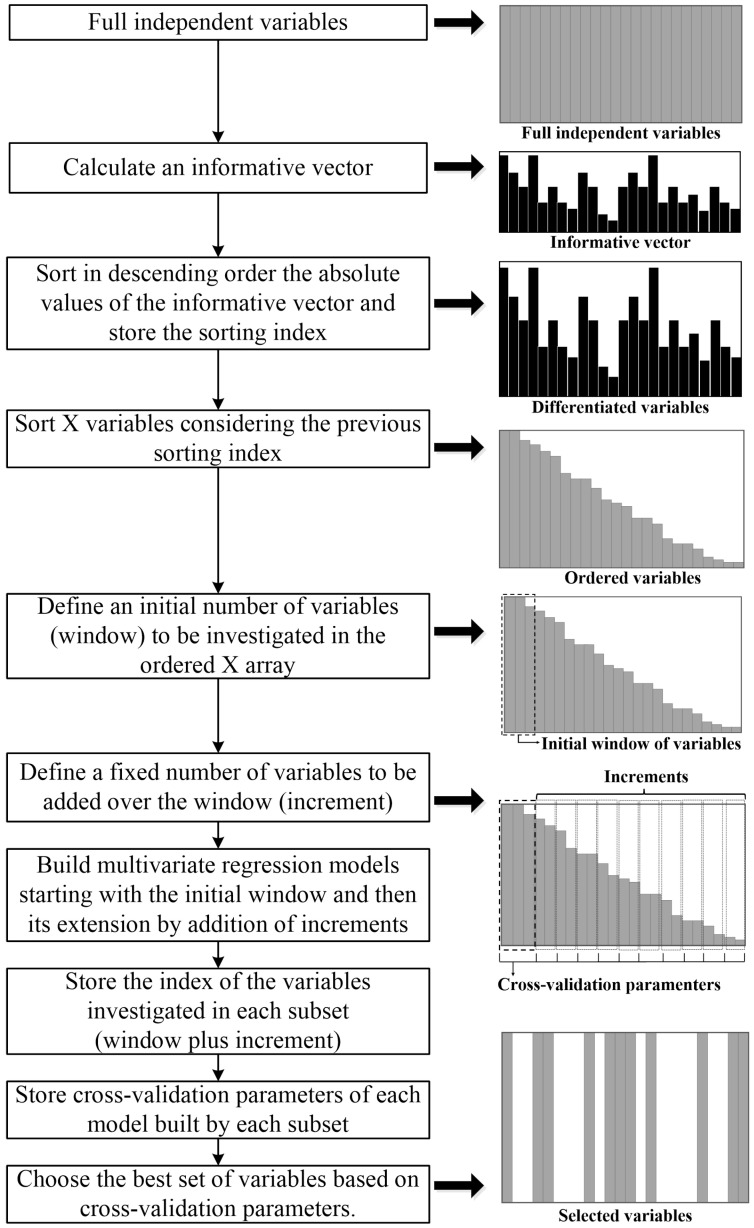
General outline of the variable selection process employing the OPS algorithm.

Three OPS methods [17], i.e., autoOPS, feedOPS and iOPS, were applied to select more informative variables to improve the models. The OPS method was performed on the basis of calculating an information vector containing information about the optimal independent variable for the prediction. The information vector was derived from the calculation involving the columns of the X matrix (independent variables) and the dependent variable (Y), with a length equal to the number of independent variables. Typically, metrics such as regression coefficient (REG), correlation of each column of the matrix X with y (COR), residual information of the reconstructed matrix with latent variables (SQR), variables importance on projection (VIP), net analyte signal (NAS), and covariance procedure (COV) are employed to compute the information vector. As depicted in Figure 3, the OPS method encompassed the following procedural steps.

In autoOPS, two new vectors were introduced, namely, the univariate regression between each column of the X matrix and y (URXY), and the vector of weights (WGHT) obtained through the NIPALS (nonlinear iterative partial least squares) algorithm. A high URXY indicates that the corresponding variable likely contains valuable information for the model. The objective of feedOPS is to incorporate feedback into the OPS algorithm, where pre-selected variables can be reintroduced into a new selection run until a specific criterion is met. OPS was applied across the entire array of segments, a strategy termed OPS interval (iOPS). Initially, the number of variables for each interval was defined, and variable selection was performed. In each interval, autoOPS or feedOPS was applied to reduce the number of variables. The intervals with only the selected variables were combined into a new matrix, and a new variable selection was conducted considering predefined windows and increments. AutoOPS (or feedOPS) was then applied to the new array to obtain the optimal set of variables.

Regarding SPA, the variable selection was constrained as the number of wavelengths selected was predetermined. One variable was randomly chosen and projected onto the rest. Variable subsets with maximum projections were formed through a sequence of projection operations. The variables in the candidate subset were used to build calibration models, and the optimal variables were determined by the root mean square error of the prediction set. Moreover, SPA could enhance modeling efficiency by judiciously selecting a subset of variables with a low degree of multi-collinearity and redundancy from the global spectrum [18].

#### 2.5.3. Multivariate Regression Models and Model Evaluation

PLS, a robust multivariate statistical tool, finds extensive application in NIR regression analysis. During the PLS quantitative modeling, the spectral matrix X and attribute concentration matrix Y were concurrently decomposed to extract latent variables (LVs). This extraction process serves to mitigate the impact of band overlap and redundant noise, effectively reducing the dimensionality of the spectral matrix and enhancing modeling performance [23].

MLR is a statistical method aimed at modeling the correlation between the involved variables and the response variable in the observed data, relying on the linear equation [24].

The model’s performance was evaluated through various statistical values, including the root mean square error of calibration (RMSEC), root mean square error of prediction (RMSEP), root mean square error of cross-validation (RMSECV), the determination coefficient (R^2^), ratio of performance to deviation (RPD), the range error ratio (RER), and relative errors RE%. Typically, an excellent model should display lower RMSE values and higher R^2^ values. Additionally, RPD was employed to assess the prediction set’s performance, representing the ratio of the standard deviation of the validation set (SD) to RMSEP. Generally, a positive correlation exists between RPD values and prediction accuracy. An RPD value less than 2 suggests an unacceptable prediction result [25]. These indices were calculated as follows:

(1) $$R^{2}=1-\frac{\sum_{i=1}^{n}{\left(y_{i}-{\hat{y}}_{i}\right)}^{2}}{\sum_{i=1}^{n}{\left(y_{i}-{\bar{y}}_{\;}\right)}^{2}}$$

(2) $$RMSE=\sqrt{\frac{1}{n}\sum_{i=1}^{n}{\left({\hat{y}}_{i}-y_{i}\right)}^{2}}$$

(3) $$RPD=\frac{SD}{RMSEP}$$

(4) $$RER=\frac{{Range}_{y}}{RMSEP}$$

(5) $$RE\;\%=\frac{y-\hat{y}}{y}\times 100\%$$

Here, *n* represents the number of samples; y signifies the reference values for GAS; $\bar{y}$ is the mean values of y; $\hat{y}$ represents the GAS values predicted by PLS regression; SD is standard deviation of the GAS reference values; ${Range}_{y}$ denotes the range of reference data.

#### 2.5.4. Software

All data pre-treatments, feature variables selection and PLS modeling were performed using the MATLAB software (ver. R2016b, The MathWorks, Natick, MA, USA). OriginPro (ver. 2020b, OriginLab, Northampton, MA, USA) was utilized for data visualization.

## 3. Results

### 3.1. Gastrodin Content

The GAS contents in *G. elata* samples are presented in Table 1. All the calibration sets (0.0531–0.1972 mg g^−1^) showed a wider range of the GAS contents than the prediction sets (0.0547–0.1806 mg g^−1^), indicating that the KS algorithm can efficiently select the representative calibration samples. The homogeneous SD values of calibration set and validation set could improve the robustness and accuracy of perdition models.

### 3.2. Spectral Interpretation

**Figure 4:**
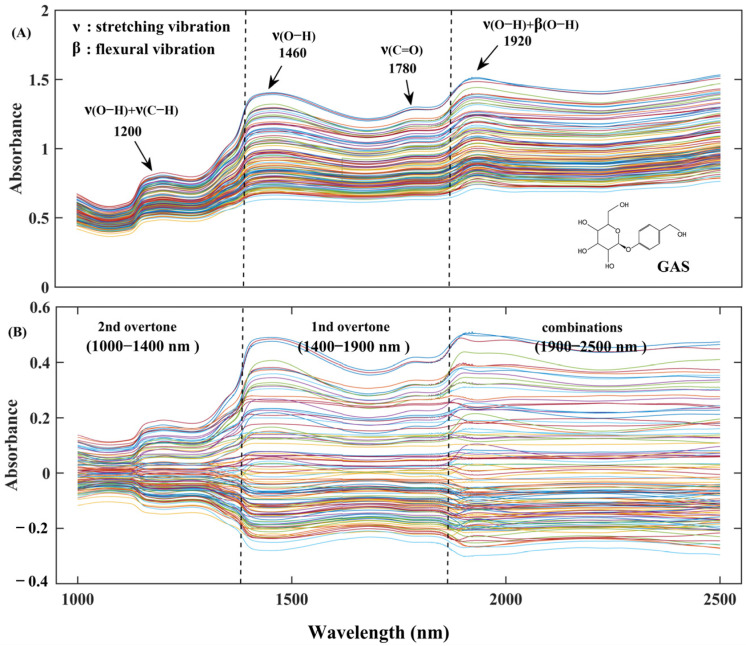
NIR spectra of *G. elata*, (**A**) raw spectra, and (**B**) mean centering transformed spectra.

The averaged FT-NIR spectra for all fresh *G. elata* samples are presented in Figure 4A). The spectra gave rise to characteristic broad and overlapping peaks between 1000 and 2500 nm. All samples presented similar spectral shapes and characteristic spectral regions, such as 1150–1250 nm, 1400–1550 nm, and 1860–2000 nm. Among these spectral signals, the specific peak around 1920 nm was mainly related to combined O-H bands of the water molecules. Energy absorption at bands 1780, 1460, 1200 nm can be highlighted, which reflected the presence of the first and second overtones of the water molecule O-H group [26]. Phenolics are compounds possessing aromatic ring with one or more hydroxyl groups, while GAS is a polyphenolic substance consisting of two aromatic rings with multiple hydroxyl groups [5]. The spectral regions between 1400 nm and 1460 nm corresponded to the first overtone of the O-H group of phenol, and 1920 nm is also the combination band of O-H vibrations [27]. According to previous reports, phenolics should be detected in the regions of 1415–1515 nm, 1650–1750 nm, and 1955–2040 nm [27,28].

### 3.3. Multivariate Analysis

#### 3.3.1. Spectral Preprocessing Methods and Modeling Based on PLSR

The full diffuse reflection spectral information of *G. elata* samples and GAS content were employed for constructing the PLSR model. Table 2 presents the outcomes of the calibration and cross-validation processes for the PLS model based on full spectra, employing various pretreatment methods. For the raw-spectrum PLS model, the developed models exhibited $R_{c}^{2}$ and $R_{cv}^{2}$ values of 0.8713 and 0.8405, respectively. The root mean square error of calibration (RMSEC) and cross-validation (RMSCV) were determined as 0.01 and 0.0111 mg g^−1^, respectively.

Given the inherent noise present in the raw spectral data, a requisite preprocessing approach was implemented to enhance the signal-to-noise ratio, thereby ensuring the development of a more precise and resilient prediction model. This section delves into the exploration of various pretreatment methods and their impact on the performance of the PLS models, as detailed in Table 2. After comparison, MC was found to be superior to the other preprocessing methods in GAS prediction, with $R_{c}^{2}$, RMSEC, $R_{cv}^{2}$, RMSECV of 0.9176, 0.008 mg g^−1^, 0.9010, and 0.0088 mg g^−1^, respectively. The utilization of the MC algorithm facilitated a streamlined interpretation of scores, enabling the derivation of confidence intervals for principal component (PC) scores. Consequently, it is strongly advised to conduct a mean-centered analysis on spectral data prior to applying multivariate data analysis methods [29]. It was imperative to exercise caution in the application of preprocessing methods, as employing an incorrect type or excessively rigorous approach could result in the inadvertent removal of invaluable information [22]. As seen in Table 2, the correlations in cross-validation sets with MSC, SNV, and 1st pretreatments were lower than that of raw spectra, which indicated that improper preprocessing methods can introduce new extraneous noise and reduce the accuracy of the PLS model. Furthermore, the utilization of MSC and SNV was predominantly directed towards mitigating the impact of sample heterogeneity arising from variations in particle size and surface scattering, as indicated by previous research [22]. However, their outcomes exhibited similarity with limited space for enhancement in the model results, and in some instances, a potential deterioration was observed. This phenomenon could be attributed to the marginal influence of particle size and surface scattering effects on the spectral characteristics of *G. elata* samples. Figure 4B presents the spectra after mean centering preprocessing in the wavelength range of 1000–2500 nm. Consequently, the spectra subjected to mean centering preprocessing were employed for subsequent variable selection and modeling analysis.

#### 3.3.2. Effects of OPS Variable Selection Methods and Modeling Based on PLSR

Drawing upon the aforementioned exposition, it is evident that FT-NIRS holds promise in predicting the GAS of *G. elata* across the entire wavelength spectrum. However, the transition from these investigations to online detection is constrained by the persisting presence of 3112 variables in the original spectra. Consequently, the imperative arose to opt for the identification of characteristic wavelengths, rather than the comprehensive wavelength range, for operational involvement while maintaining comparable accuracy. The performance metrics of the PLS model based on the complete set of variables (Full) and the subset of selected variables (autoOPS, FeedOPS, and iOPS) are detailed in Table 3.

As depicted in Table 3, the PLS regression model using OPS presented a higher R^2^ (>0.95) and a lower RMSE value than the full model, which indicated that models built by removing non-informative variables would produce better prediction results compared to high-dimensional data [17]. However, the utilization of autoOPS for variable selection resulted in a reduced RMSEP, signifying enhanced predictive capabilities compared to models employing feedOPS and iOPS. The autoOPS-PLS model demonstrated notable predictive parameters: $R_{c}^{2}$ = 0.9656, RMSEC = 0.0052 mg g^−1^, $R_{p}^{2}$ = 0.9413, RMSEP = 0.0074 mg g^−1^, and RPD = 3.70. Oliveira et al. also highlighted that the autoOPS algorithms coupled with PLS models achieved a satisfactory prediction performance for the oil content in Macaw fruits by FT-NIRS [13]. In accordance with this, variable selection optimization was conducted through ordered predictors selection (OPS), yielding streamlined and predictive PLS calibration models.

**Figure 5:**
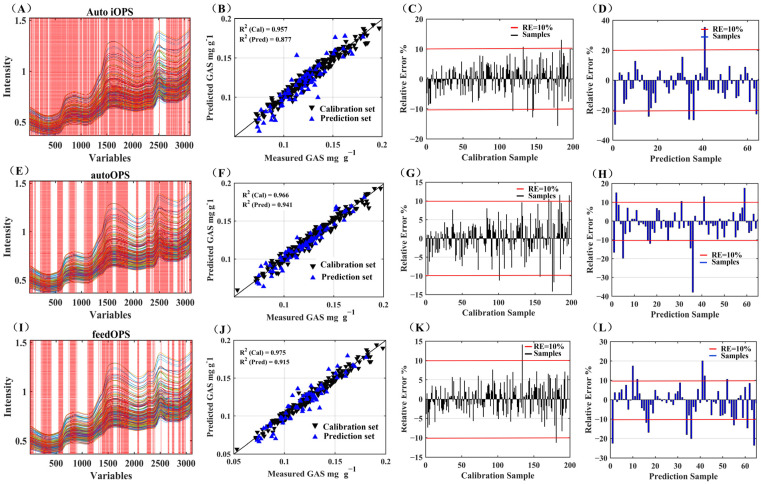
Results of spectral variables selection by different OPS approaches (**A**,**E**,**I**), the red dashed line represents the selected variable; measured and predicted GAS content using different OPS methods (**B**,**F**,**J**) (▼ represents the calibration set and ▲ represents the prediction set); relative error for calibration (**C**,**G**,**K**) and prediction (**D**,**H**,**L**).

Figure 5 presents the measured versus predicted GAS content values (Figure 5B,F,J) using the FT-NIR spectra for the same dataset based on different OPS algorithms. A linearly fitted scatter plot illustrates the calibration and prediction datasets, affirming the PLS model’s proficiency in accurately estimating the GAS content in fresh *G. elata*. Notably, Mariani et al. conducted a study on jaboticaba fruit, determining total anthocyanin content via NIRS, with correlation coefficients between predicted and measured values ranging from 0.65 to 0.89 [30]. However, concerning GAS content, our model exhibits superior parameters, substantiating the feasibility of predicting GAS content through FT-NIR spectroscopy.

The selected variables for GAS were obtained by iOPS (Figure 5A), autoOPS (Figure 5E) and feedOPS (Figure 5I). The variables selected using the three OPS algorithms were similarly distributed at 2nd overtone, 1st overtone and combination regions. However, autoOPS selected more variables in the combination region, especially in the interval of 1800 to 2200 nm associated with stretching vibration and bending vibration of O-H [31]. Indications point towards the combination region harboring substantial information conducive to GAS prediction. The application of autoOPS emerges as a universally robust variable screening method, demonstrating its efficacy in significantly enhancing the predictive capabilities of the model.

In Figure 5, the relative errors of calibration are depicted in panels C, G, and K, while the relative errors of prediction are illustrated in panels D, H, and L. Notably, a majority of the relative errors observed in Figure 5G,F are below 10%, affirming the capability of the autoOPS-PLS model to effectively predict the GAS content in *G. elata*.

#### 3.3.3. Multiple Linear Regression at Selected Wavelengths Based on SPA

Although the autoOPS algorithm has been proven to select more informative and predictive variables, a total of 1305 variables were still retained, which was not conducive to interpreting the spectra, saving computational time and reducing hardware costs. To this end, we used the SPA algorithm to further implement the screening of effective bands.

After determining the optimal wavelength based on the SPA results, the corresponding MLR and PLS models were developed. The MLR technique offers a more straightforward mathematical interpretation in scenarios involving low-dimensional data, in contrast to the PLS model. Consequently, it demands minimal analytical knowledge [32]. Statistical parameters for the PLS model and MLR model using selected variables are shown in Table 4.

**Figure 6:**
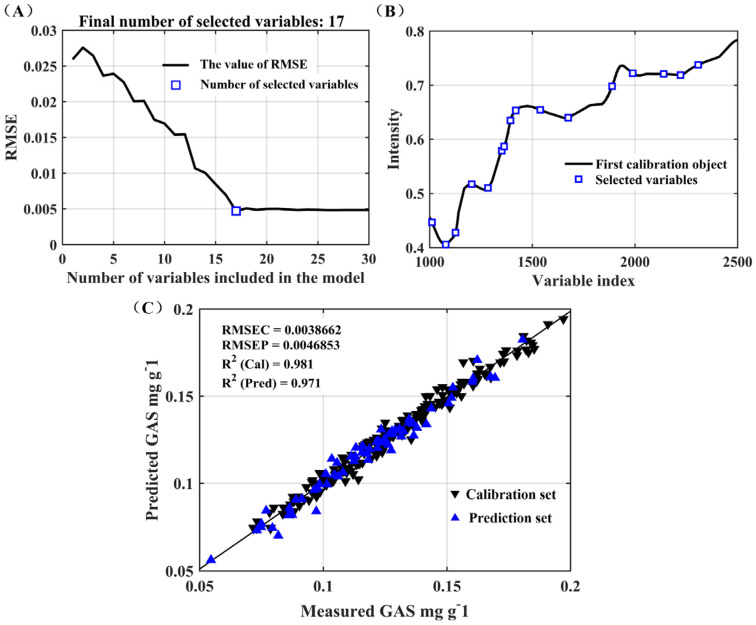
(**A**) A curve illustrating the RMSE; (**B**) distributions of characteristic variables using SPA; and (**C**) scatter plots depicting the correlation between measured and predicted GAS values utilizing MLR with variables selected by SPA (▼ represents the calibration set and ▲ the prediction set).

Table 4 presents the results of the MLR modeling, employing 17 variables selected through SPA. The optimal performance was realized with $R_{c}^{2}$ = 0.9808, RMSEC = 0.0039 mg g^−1^, $R_{p}^{2}$ = 0.9712, and RMSEP = 0.0047 mg g^−1^. Both RPD and RER were utilized as parameters for the precision assessment of the models. It is generally considered that an RPD value higher than 2 indicates a highly accurate prediction model [33]. In addition, some studies require the RMSEP to be greater than RMSEC [34]. In contrast to the PLS model, the autoOPS-SPA-MLR model exhibited superior predictive capabilities, evident in its RPD of 5.83 and the RMEP/REMEC standing at 1.2. The graphical representation in Figure 6A,B depicts the progression of RMSE with the increase in the selection of variables and illustrates the distributions of characteristic variables as determined by the SPA method. It was obvious that RMSE had a downward trend and remained stable after *n* > 17. The optimal sample subsets contained 17 feature variables for GAS. The selected wavelength points are listed in Table 4. Specifically, wavelengths at 2140, 1959, and 1011 nm corresponded to the combination band and the second overtone of phenol O-H stretching vibrations. This observation underscores the efficacy of employing autoOPS-SPA for the development of a robust model.

Several previously conducted studies were comparatively analyzed to further assess the conclusions drawn in this investigation. For instance, Li et al. utilized near-infrared spectroscopy in conjunction with PLSR to ascertain the total flavonoids and saponins contents in bottled Compound E Jiao oral liquid [10]. The optimal outcomes included RMSEP = 0.0844 and $R_{p}^{2}$ = 0.9622 for total flavonoids, and RMSEP = 0.0139 and $R_{p}^{2}$ = 0.9801 for saponins. In comparison, the findings in our study for GAS were notably more satisfactory, featuring an $R_{p}^{2}$ of 0.9843 and an RPD of 7.46. Similarly, Lei et al. quantified several components in *Angelica sinensis* utilizing the same spectroscopic method. The regression models in their study provided robust predictions for chlorogenic acid and ferulic acid, achieving $R_{p}^{2}$ values exceeding 0.90 [34]. However, the performance of our GAS model in this work surpassed these results. Comparative analysis with outcomes from other studies on various plants or traditional Chinese medicine reveals that the quantitative performance of our study on GAS generally falls within a reasonable and commendable range.

## 4. Discussion

The results confirmed the potential of NIRS for the rapid and reliable assessment of GAS in fresh *G. elata* in a rapid and nondestructive manner. However, the analysis of Traditional Chinese Medicine (TCM) by NIRS poses greater challenges due to the presence of numerous components at low concentrations. The amount of each effective or potentially effective chemical component that has been identified is often less than the lower analytical limit of NIRS, which greatly limits the application of NIRS to TCM analysis [35]. The reduced sensitivity of NIR poses calibration challenges, particularly when dealing with low concentrations of a component of interest (typically below 0.1%). Despite this, NIRS demonstrates acceptable accuracy in quantitatively analyzing trace active ingredients in agricultural products and herbal medicines, thereby meeting quality control requirements. Noteworthy examples include the determination of lutein and β-carotene in cabbage [36] and pinene, methyl salicylate, and eugenol in safflower oil [36], all yielding satisfactory results through NIRS. Furthermore, the occurrence of indirect determination of one or more parameters through correlations between variables is plausible. In this study, the PLS and MLR calibration models employed may be influenced by another component of the GAS, serving as a covariate, such as moisture. In such instances, NIR spectroscopy becomes a valuable tool for the indirect measurement of its concentration [37]. Specifically, the best predictions were obtained when considering a combination of both variable selection methods, with an $R_{c}^{2}$ of 0.9808 and an $R_{p}^{2}$ of 0.9712 (autoOPS-SPA-MLR).

Currently, prominent wavelength selection methods in multivariate calibration analysis encompass uninformative variable elimination (UVE), random frog (RF), competitive adaptive reweighted sampling (CARS), and interval partial least squares regression (iPLS). In our preliminary assessments, these variable screening methods were systematically evaluated and compared with OPS. Notably, PLS calibration models based on autoOPS-screened variables demonstrated superior performance. Compared with the full-spectrum modeling approach, the SPA-MLR method is constructed from a smaller subset of variables, which is more concise and easier to interpret. In some applications, the SPA-MLR method provides better prediction results than the PLS method [38].

In conclusion, the stability of the model dictates the accuracy of quantitative analysis by NIRS. It is influenced by a series of factors such as the accuracy of chemical reference value determination, the number of spectra representative of the model, equipment factors and human factors. A sufficient number of representative samples, strict control of experimental conditions and adoption of appropriate data-processing methods all impact on chemical reference values and accuracy.

## 5. Conclusions

Because the traditional HPLC method for determining GAS in fresh *G. elata* is highly time consuming, the potential of FT-NIRS combined with several spectral preprocessing and variable selection methods to rapidly and accurately determine the GAS content in fresh *G. elata* was investigated. After comparison, MC was found to be the optimal pre-processing method. The variable selection methods, especially autoOPS, could optimize the PLS model performances by selecting more predictive and informative variables. The best calibration model established using autoOPS-SPA-MLR was obtained for GAS prediction with a high prediction capacity ($R_{p}^{2}$ = 0.9712, RPD = 5.83 and RER = 27.65). The method presented was faster, nondestructive, solvent-free, and low-cost than the reference methods.

## Figures and Tables

**Table 1:** Statistics of the GAS contents in mg g^−1^ of all *G. elata* samples.

Dataset	Sample Size	Range	Mean ± SD
Calibration	197	0.0531–0.1972	0.1276 ± 0.0279
Prediction	65	0.0547–0.1806	0.1171 ± 0.0274

Abbreviation: SD—standard deviation.

**Table 2:** Calibration and cross-validation results for GAS constructed utilizing PLSR models based on FT-NIRS information with different pretreatment methods.

Pretreatment	LVs	Calibration Set		Cross-Validation Sets	
		$R_{c}^{2}$	RMSEC	$R_{cv}^{2}$	RMSECV
Raw	10	0.8713	0.010	0.8405	0.0111
MC	10	0.9176	0.008	0.9010	0.0088
Autoscale	10	0.9168	0.0079	0.9002	0.011
SG	10	0.8576	0.0105	0.8276	0.0158
1st Derivative	10	0.9355	0.0071	0.8711	0.01
MSC	10	0.8817	0.0096	0.8496	0.011
SNV	10	0.8818	0.0096	0.8495	0.011

Abbreviations used in this context include LVs, denoting the number of latent variables; RMSEC and RMSECV, which represent the root mean square errors of calibration and cross-validation, respectively; and $R_{c}^{2}$ and $R_{cv}^{2}$, indicative of the determination coefficients for calibration and cross-validation, respectively.

**Table 3:** Calibration and prediction results for GAS developed using PLSR models based on the FT-NIRS information with OPS variable selection methods.

Methods	LVs	nVars	Calibration Set		Prediction Set		RPD	RER	SEP/SEC
			$R_{c}^{2}$	RMSEC	$R_{p}^{2}$	RMSEP			
Full	10	3112	0.9176	0.008	0.8363	0.0135	2.03	9.63	1.69
autoOPS	10	1305	0.9656	0.0052	0.9413	0.0074	3.70	17.57	1.42
FeedOPS	8	730	0.9748	0.0044	0.9148	0.0091	3.01	14.29	2.07
iOPS	10	1140	0.9574	0.0058	0.8772	0.0115	2.38	11.30	1.98

Abbreviation: LVs represents the count of latent variables, while nVars indicates the total number of variables. RMSEC is the root mean square error of calibration, measuring the precision of the calibration model, with corresponding determination coefficients denoted as $R_{c}^{2}$; RMSEP stands for the root mean square error of prediction, accompanied by determination coefficients $R_{p}^{2}$; RPD: residual predictive deviation; SEP/SEC signifies the ratio of RMSEP to RMSEC, offering insights into model reliability; RER, or range error ratio, encapsulates the ratio of prediction range to actual values.

**Table 4:** Calibration and prediction results for GAS developed using PLS and MLR models based on FT-NIRS information.

Model	Method	nVars	$R_{c}^{2}$	RMSEC	$R_{p}^{2}$	RMSEP	RPD	RER
PLSR	autoOPS-SPA	1011, 1078, 1125, 1200, 1283, 1351, 1362, 1393, 1420, 1538, 1675, 1888, 1959, 2140, 2221, 2307, 2500 nm	0.9235	0.0077	0.8904	0.0096	2.85	13.54
MLR			0.9808	0.0039	0.9712	0.0047	5.83	27.65

Abbreviation: nVars—number of variables; RMSEC—root mean square errors of calibration; $R_{c}^{2}$—determination coefficients of calibration; RMSEP—root mean square errors of prediction; $R_{p}^{2}$—determination coefficients of prediction; RPD—residual predictive deviation; RER—range error ratio.

## Data Availability

The data presented in this study are available on request from the corresponding author.

## Supplementary Materials

The following supporting information can be downloaded at https://www.mdpi.com/article/10.3390/foods12244435/s1. Figure S1: The RGB image of *G. elata*.; Figure S2: HPLC chromatogram of *G. elata* samples (No.1–50).
