# Supplementary material for: A Novel Variable Selection Method Based on Ordered Predictors Selection and Successive Projections Algorithm for Predicting Gastrodin Content in Fresh Gastrodia elata Using Fourier Transform Near-Infrared Spectroscopy and Chemometrics
Source: Foods. 2023 Dec 11;12(24):4435. doi: 10.3390/foods12244435 (PMC10743185; doi:10.3390/foods12244435)
Supplement: Supplementary file 1 [file foods-12-04435-s001.zip › foods-2741213-supplementary.pdf]

Supplementary Materials

Figure caption:

Figure S1. The RGB image of *G. elata.*

Figure S2. HPLC chromatogram of *G. elata* samples (No.1-50).

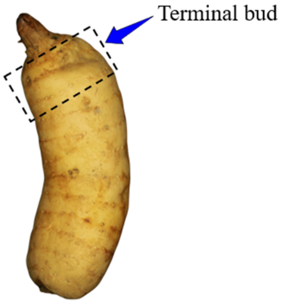

Figure S-1

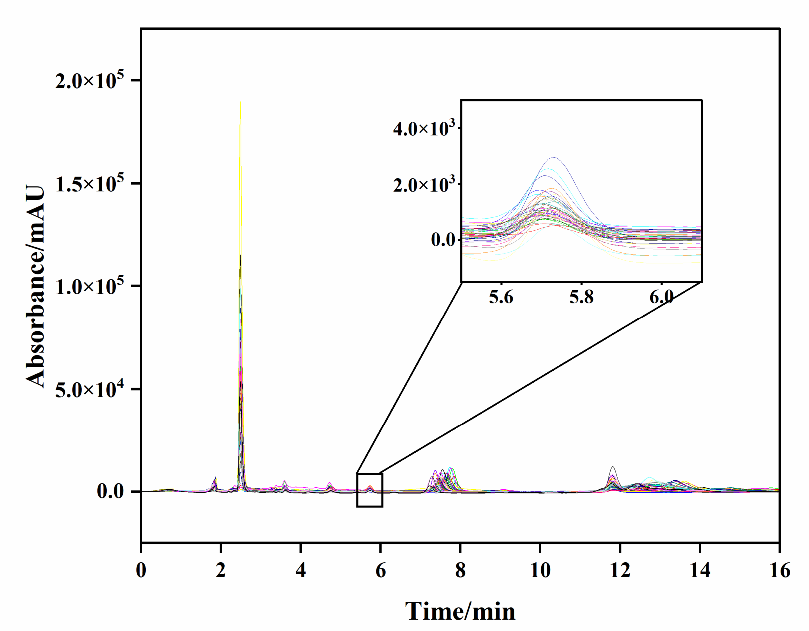

Figure S-2
